# Pioglitazone treatment prior to transplantation improves the efficacy of human mesenchymal stem cells after traumatic brain injury in rats

**DOI:** 10.1038/s41598-019-49428-y

**Published:** 2019-09-20

**Authors:** Mahasweta Das, Karthick Mayilsamy, Xiaolan Tang, Jung Yeon Han, Elspeth Foran, Alison E. Willing, Shyam S. Mohapatra, Subhra Mohapatra

**Affiliations:** 1 James A. Haley Veterans Hospital, University of South Florida College of Medicine, Tampa, FL 33612 USA; 2 Department of Molecular Medicine, University of South Florida College of Medicine, Tampa, FL 33612 USA; 3 Department of Internal Medicine, University of South Florida College of Medicine, Tampa, FL 33612 USA; 4 Department of Neurosurgery and Brain Repair, University of South Florida College of Medicine, Tampa, FL 33612 USA

**Keywords:** Neurodegeneration, Mesenchymal stem cells

## Abstract

Traumatic brain injury is a leading cause of death and disability around the world. So far, drugs are not available to repair brain damage. Human mesenchymal stem cell (hMSC) transplantation therapy is a promising approach, although the inflammatory microenvironment of the injured brain affects the efficacy of transplanted hMSCs. We hypothesize that reducing the inflammation in the cerebral microenvironment by reducing pro-inflammatory chemokines prior to hMSC administration will improve the efficacy of hMSC therapy. In a rat model of lateral fluid percussion injury, combined pioglitazone (PG) and hMSC (combination) treatment showed less anxiety-like behavior and improved sensorimotor responses to a noxious cold stimulus. Significant reduction in brain lesion volume, neurodegeneration, microgliosis and astrogliosis were observed after combination treatment. TBI induced expression of inflammatory chemokine CCL20 and IL1-β were significantly decreased in the combination treatment group. Combination treatment significantly increased brain-derived neurotrophic factor (BDNF) level and subventricular zone (SVZ) neurogenesis. Taken together, reducing proinflammatory cytokine expression in the cerebral tissues after TBI by PG administration and prior to hMSC therapy improves the outcome of the therapy in which BDNF could have a role.

## Introduction

Traumatic brain injury (TBI) is a leading cause of death and disability in the United States and around the world. Approximately 10 million people globally either are hospitalized or die from TBI each year^1^. In the United States alone 1.7 million people suffer from TBI and about 52,000 people die^2^. It affects both children and adults and affects the quality of life of the survivors, posing a significant social burden. The most common causes of TBI are related to motor vehicle accidents, sports and battlefield injuries. Falls, especially in children and older people, and physical abuse are among the common causes as well. The National Center for Injury Prevention and Control in the United States recorded 715.7 per 100,000 people TBI-related emergency department visits, 91.7 hospitalizations and 17.1 deaths in 2010^3^ with an approximate annual financial burden of $76.5 billion^3^. TBI affects the quality of life of the survivors. Even mild TBI or concussions may have effects later in life that are difficult to relate to the prior TBI event. The primary insult to the brain damages the cerebral tissues and the BBB and evokes immune response systemically and locally in the brain. This leads to astrogliosis, immune cell migration, cytokine and chemokine secretion and microglial activation. All these immunological reactions cause further damage to the brain and the secondary injury spreads over time, inducing long term structural, functional, behavioral and psychological deficits.

The available drugs only provide symptomatic treatments to TBI patients. Clinical trials of erythropoietin or progesterone failed to repair brain damage^4–6^. Transplantation of mesenchymal stromal cells (MSCs) has been shown to be the most promising regenerative approach. These pluripotent cells have the potential to be transformed into many different cells, are immune suppressive and secrete growth factors to promote tissue regeneration^7–9^. In addition to time of transplantation, number and quality of transplanted cells, the efficacy of MSC therapy largely depends on the microenvironment of the target tissue. Following TBI, proinflammatory cytokines are secreted in the brain parenchyma, immune cells migrate and glial cells are activated creating an inflammatory environment which may not favor the survival and functioning of the MSCs^10^. Therefore, reducing the inflammation before MSC therapy might help to increase the efficacy of the therapy.

We hypothesize that reducing proinflammatory cytokine production in brain tissue will enhance the efficacy of hMSC treatment and improve the histopathological and behavioral outcomes in rat after TBI. Previously we have shown that the proinflammatory chemokine CCL20 is produced in the degenerating cerebral tissues after TBI^11,12^. Pioglitazone is a peroxisome proliferator-activated receptor gamma (PPARγ) agonist belonging to the thiazolidinedione (TZD) class of drugs and used to treat hyperglycemia. When used in low dose it showed a cardio-protective property in experimental animals^13^. Qi *et al*. (2011) have shown that PPARγ negatively regulates CCL20 expression in the renal epithelium^14^. PG has also been shown to reduce proinflammatory cytokines IL1-β and IL-6 in different tissues^15–17^. Therefore, in this study we chose to use PPARγ agonist pioglitazone (PG) to reduce TBI-induced cytokine production in the cerebral tissues and thereby, improve the microenvironment for hMSC functioning after TBI.

## Results

### Intra-nasally administered hMSCs were delivered to the brain post TBI

**Figure 1:**
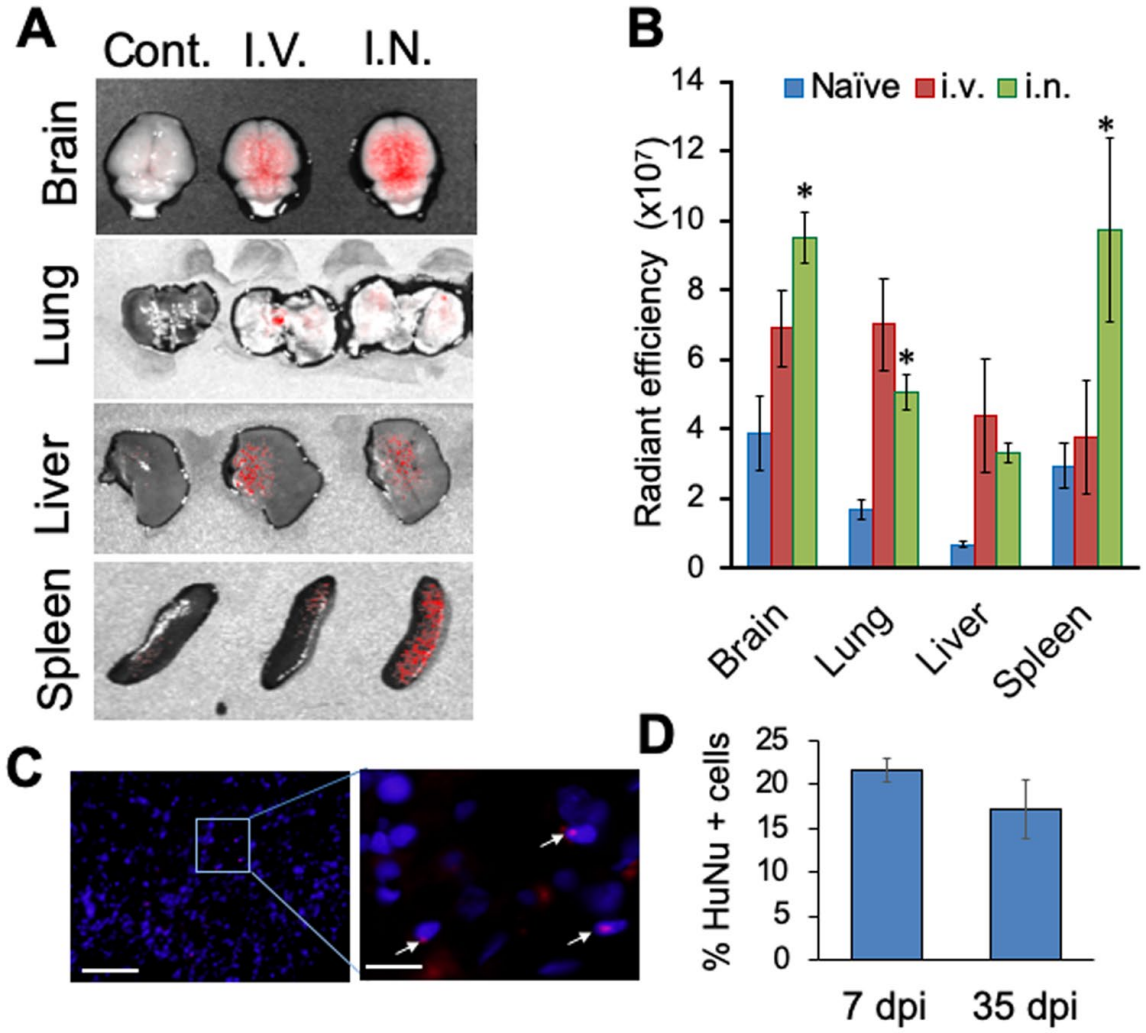
Delivery of hMSC after TBI. (**A**) Biodistribution of hMSCs after TBI. Representative Xenogen images showing DiR fluorescence in different organs 7 days after TBI. (**B**) Histogram showing the significantly higher level of DiR fluorescence in brain and spleen following intra-nasal (i.n.) administration as compared to intra-venous (i.v.) administration, n = 3 animals/group. *p < 0.01 vs i.v. (**C**) Brain sections from rats after 35 days of TBI were stained with anti-human nuclear antigen (HuNu) antibody (clone 235-1). The immunostaining indicates the presence of hMSCs (arrows) in the ipsilateral cortex close to the lesion site. Scale bar 100 µ, inset scale bar 20 µ. (**D**) Histogram showing the percentage of HuNu positive cells in the perilesional area of the brains. HuNu positive cells which were also DAPI positive were counted from the perilesional area of each section located 120 µ apart. Also, 50 DAPI positive cells were counted from the same area of the sections. % of HuNu positive cells was calculated from 7 dpi and 35 dpi brains as shown in the histogram. A paired t test did not show any significant difference between the % of HuNu positive cells present in the brains of 7 dpi or 35 dpi. n = 3 (7 dpi), and 7 (35 dpi).

**Figure 2:**
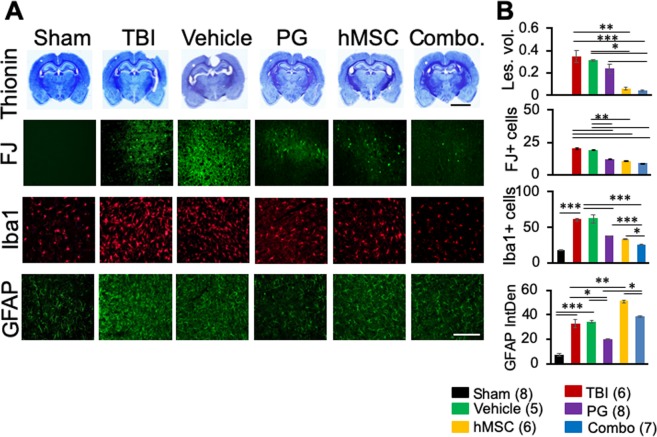
hMSC or PG + hMSC combination treatments reduce brain damage and neuroinflammation 35 days after TBI. Representative images of brain sections (**A**) or histograms showing image J quantitation. (**B**) Thionin images are low magnification images (scale bar 500 µ), showing tissue loss. The average tissue loss is expressed as lesion volume (mm^3^) in the histogram. Remaining panels in A show degenerating cells and fibers (FJ panel), microgliosis (Iba1 panel) or astrogliosis (GFAP panel) in the ipsilateral cortex 35 days after TBI. Lesion volume was measured in mm^3^, FJ and Iba1 were measured by counting positive cells and GFAP was measured by integrated density using image J. Les vol., lesion volume, PG, pioglitazone, hMSC, human mesenchymal stem cells, combo., PG + hMSC combination treatment. Numbers in the parenthesis indicate the number of animals in each group. *p < 0.01, **p < 0.001 vs TBI, ***p < 0.0001. Scale bar 100 µ.

Intranasal (i.n.) delivery of hMSCs to the brain was confirmed in a subset of rats. DiR labeled hMSCs were delivered through i.n. or intravenous (i.v.) routes. IVIS imaging showed the presence of DiR fluorescence in brain, lung, liver and spleen 7 days after hMSC administration (Fig. 1A,B). The i.n. delivery showed significantly higher DiR signal in the brain compared to i.v. delivery (Fig. 2B). DiR fluorescence indicates that a large portion of the hMSCs delivered by i.v. route was cleared by the system in 7 days, whereas, the i.n. delivered hMSCs stayed in the system. Presence of hMSCs in the brain 35-days post impact (dpi) was confirmed by immunohistochemical staining of the brain sections with anti-human nuclear antigen (clone 235-1) antibody (HuNu). The immunostaining showed the existence of HuNu positive nuclei in the ipsilateral cortex close to the lesion area in hMSC or combination treated rats (Fig. 1C). In addition, a few scattered HuNu-DAPI double positive cells were observed in the treated brains. The number of HuNu positive cells in the perilesional area of the brains observed at 7 dpi did not change significantly at 35 dpi (Fig. 1D).

### Combination treatment reduced the pathological changes and neurodegeneration in the rat brain cortex

Thionin staining clearly shows tissue loss and decreased cellularity in the TBI and vehicle treated animals as compared to sham. Significant cortical tissue loss was evident after 35 dpi. Lesion volume in the vehicle treated group was not significantly different from the TBI alone group. PG treated animals showed some reduction in the lesion volume but this was not statistically significantly different from lesion volume in the TBI alone group. On the other hand, lesion volume decreased significantly after hMSC or combination treatment (Fig. 2A,B).

Neurodegeneration is evident in the TBI animals 35 dpi. FJ staining in the rat brain shows damaged FJ positive cells and fibers in the ipsilateral cortex around the impact site and the lateral part of the cortex, around the primary sensorimotor area in TBI and the vehicle treated groups. White matter damage was also visible in these brains in the ipsilateral corpus callosum and striatum. The treated groups (PG, hMSC or combination) showed significantly fewer degenerating cells or fibers than the TBI or vehicle groups, but there was no significant difference among the treatment groups (Fig. 2A–FJ panel, B). FJ positive fibers were also observed in corpus callosum in the regions adjacent to the degenerated areas in TBI or vehicle treated groups. Some FJ positivity was also observed in the PG or hMSC treated animals but not in the combination treated group (Fig. S1). Contralateral brain did not show cellular or fiber damage in animals from any of the groups.

### Effect of combination treatment on neuroinflammation

#### Microgliosis

Microgliosis, as indicated by an increased number of Iba1 positive microglia in the cerebral tissue, was observed 35 dpi in TBI, as well as vehicle treated animals. The number of Iba1 positive microglia significantly increased in TBI animals as compared to sham. Increased number of microglia were observed in the ipsilateral cortex (adjacent to the injury site) (Fig. 2 A-Iba1 panel, B-Iba1 + cells), lateral part of the cortex and corpus callosum (Fig. S1A). All the treatment groups showed significantly fewer Iba1 positive microglia in these areas compared to TBI or vehicle treated groups in the cortex, and the combination treatment group showed significantly fewer Iba1 positive cells compared to TBI, vehicle or PG treated animals (Fig. 2 B-Iba1 + cells).

The number of Iba1 positive cells also increased in the contralateral hemisphere of these areas but not as much as the corresponding areas of the ipsilateral hemisphere (Fig. S2). In the contralateral corpus callosum also, combination treated animals showed significantly fewer Iba1 positive cells as compared to TBI, vehicle, PG or hMSC groups.

#### Astrogliosis

Increased GFAP immunoreactivity with scar formation was observed in all other groups as compared to sham (Fig. 2A- GFAP panel, B-GFAP integrated density) indicating activation of astrocytes 35 dpi. In the TBI and vehicle groups, astroglial activation and glial scars were still observed after 35 dpi. PG treatment reduced the GFAP immunoreactivity significantly as compared to TBI or vehicle treated animals. In hMSC treated animals, the GFAP immunoreactivity increased significantly compared to TBI, vehicle or PG treated groups, although close observation did not reveal glial scar formation in these animals. In the combination treated animals, GFAP immunoreactivity was high, although not significantly, compared to the TBI or vehicle groups, and no glial scars were observed in these brain sections. On the other hand, in this group, GFAP immunoreactivity was significantly reduced from the hMSC treated group (Fig. 2B-GFAP integrated density).

### Cytokine expression

**Figure 3:**
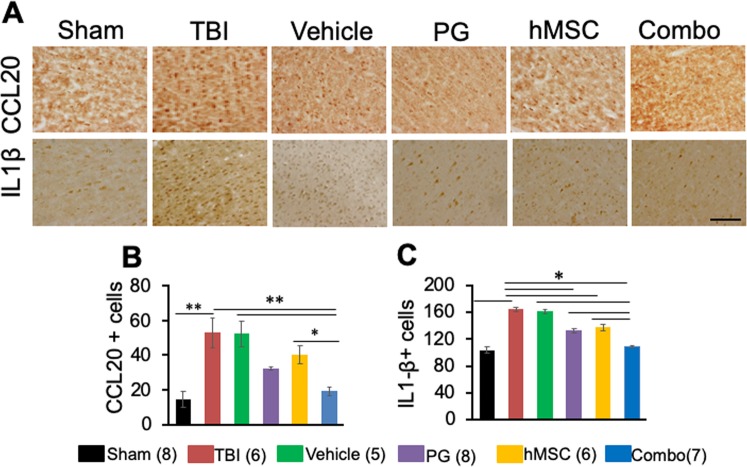
Combination treatment significantly decreases the expression of inflammatory chemokines CCL20 and IL1-β in the rat brain cortex 35 days after TBI. (**A**) Representative images of immunoperoxidase staining of cortex (adjacent to the injury site) showing the CCL20 or IL1-β immunoreactivity under different experimental conditions. Scale bar 100 µ. (**B**,**C**) Histograms showing the numbers (mean ± SEM) of CCL20 (B) or IL1 β (**C**) positive cells in the cortex. PG, pioglitazone, hMSC, human mesenchymal stem cells, combo., PG + hMSC combination treatment. Numbers in parentheses indicate the number of animals in each group. *p < 0.01, **p < 0.001.

Expression of CCL20 is a landmark of neuroinflammation after TBI. After 35 dpi, TBI and vehicle treated animals express significantly more CCL20 in the cortex compared to sham (Fig. 3A,B). In our pilot study, we observed that CCL20 expression was up-regulated and PPARγ was downregulated in the brain 48 h post-TBI (Fig. S3). Also, PG treatment reduced CCL20 expression and increased PPARγ expression 48 h post TBI (Fig. S3). In the present study, PG treatment reduced CCL20 expression 35 dpi. Both hMSC treatment, as well as combination treatment, reduced CCL20 expression in the cortex. Importantly, CCL20 expression in the ipsilateral cortex in the combination treatment group was significantly lower than that in hMSC treatment group indicating better efficacy of the combination treatment (Fig. 3A,B). The expression of IL1-β in the ipsilateral cortex was also increased in the TBI or vehicle treated groups and decreased after PG, hMSC or combination treatment. IL1-β expression decreased significantly in the combination treated group compared to hMSC treated group (Fig. 3A,C). This cytokine expression profile clearly indicates that PG treatment prior to hMSC therapy reduced the proinflammatory microenvironment facilitating the performance of hMSC.

### PG-hMSC combination treatment increased neurogenesis

**Figure 4:**
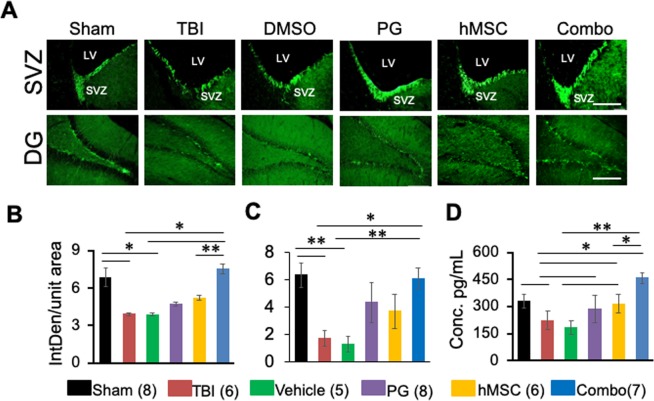
PG-hMSC combination treatment improves neurogenesis and increases BDNF level after TBI. (**A**) Representative immunofluorescence images showing DCX immunoreactivity in the SVZ (Scale bar 100 µ) or DG region (Scale bar 200 µ) under different experimental conditions. (**B**) Histogram showing DCX immunoreactivity (Mean ± SEM) in SVZ (**B**) or DG (**C**) under different experimental conditions measured using image J. (**D**) Histogram showing serum BDNF level measured by ELISA. IntDen, integrated density, PG, pioglitazone, hMSC, human mesenchymal stem cells, SVZ, subventricular zone, LV, lateral ventricle, DG, dentate gyrus. Numbers in parentheses indicate the number of animals in each group. *p < 0.01, **p < 0.001.

Neurogenesis is an important indicator of recovery from TBI. Neurogenesis occurs at a basal level in the adult, uninjured animals (sham) in the sub-ventricular zone (SVZ) area of the brain as observed in our experiment. In TBI or vehicle treated animals, neurogenesis significantly decreased as indicated by decreased DCX immunoreactivity. PG or hMSC treatments increased neurogenesis but not significantly compared to TBI or vehicle treated animals, whereas combination treatment significantly increased neurogenesis in the SVZ region compared to TBI or vehicle groups. In the combination group, neurogenesis was restored to the same extent observed in sham animals (Fig. 4A,B).

### PG-hMSC combination treatment increased brain-derived neurotrophic factor (BDNF) secretion

The neurotrophic factor BDNF supports neurogenesis. We observed that serum BDNF level, as measured by ELISA, decreased in TBI and vehicle treated groups. PG treatment restored the level close to sham animals while hMSC and combination treatment significantly increased serum BDNF level. BDNF level after combination treatment was significantly higher than in the hMSC treated group (Fig. 4C).

### Combination treatment reduced the anxiety-like behavior of TBI rats in the open field

#### Behavior in the Center Zone (CZ)

**Figure 5:**
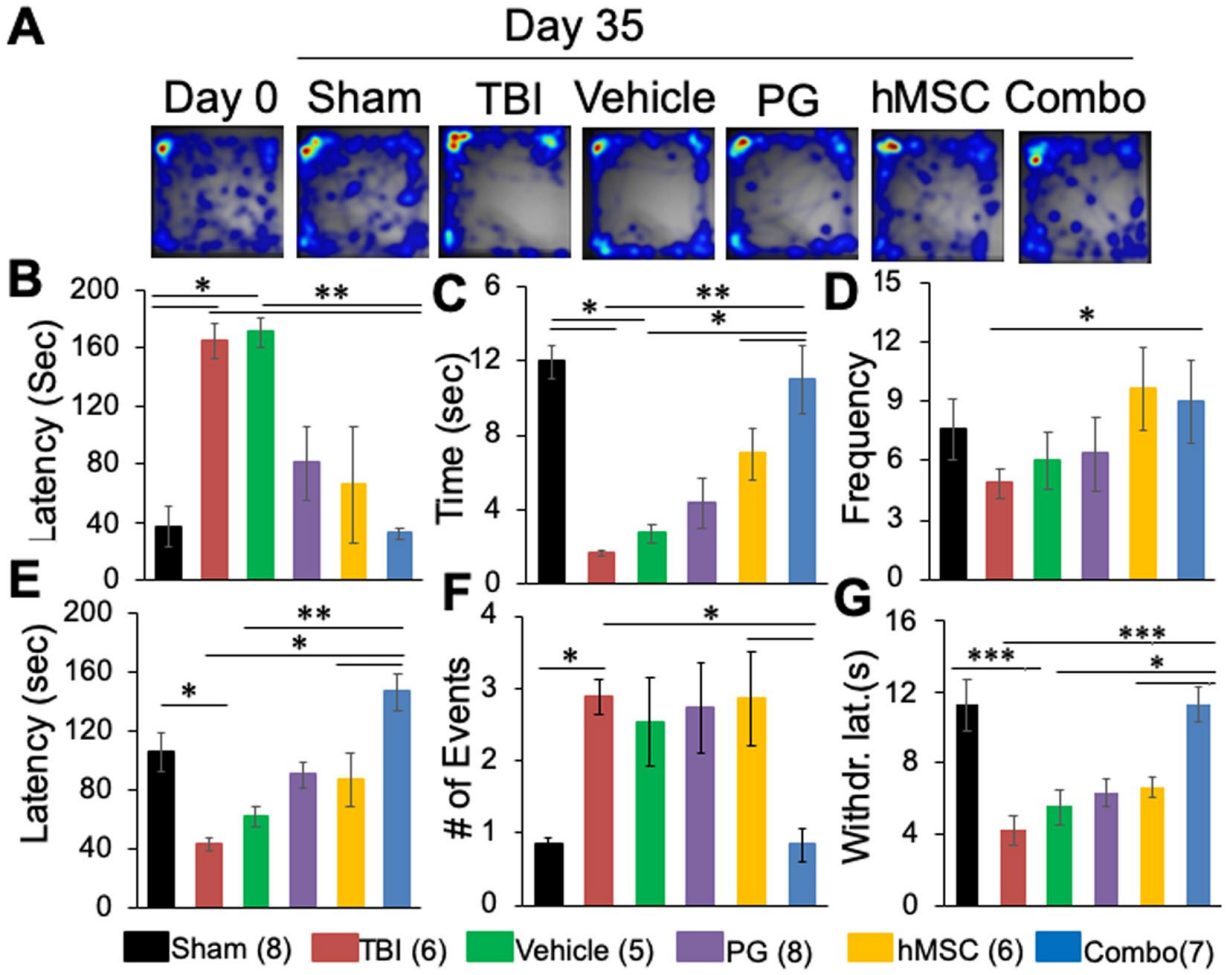
Open field or sensorimotor behavior of rats under different experimental conditions. (**A**) Representative heat maps of the movement of rats in the open field arena. Baseline behavior (Day 0) was recorded before TBI or sham surgeries. Histograms show the average values (mean ± SEM) of anxiety-like behaviors of rats in the open field arena. (**B**–**F**) Anxiety-like behavior of rats in the open field, (**B**) Latency to the first entry to the center zone of the arena, (**C**) Time spent in the center zone of the arena, (**D**) Frequency to entry to the center zone, (**E**) Latency to first immobility in the arena, (**F**) Number of grooming events. (**G**) Mean paw withdrawal latency (sec) of the contralateral paw to noxious cold stimulation (mean ± SEM). PG, pioglitazone, hMSC, human mesenchymal stem cells, Combo, PG + hMSC combination treatment. Numbers in parentheses indicate the number of animals in each group. *p < 0.05, **p < 0.01, ***p < 0.001.

Rat behavior in the open field arena before and after TBI was recorded and analyzed using Noldus Ethovision software as described in the methods section. A heat map was generated showing the activities in the arena (Fig. 5A). As a general tendency, rats preferred to stay close to the walls of the apparatus. The baseline (Day 0) behavior showed that the rats were visiting the CZ frequently while staying mostly to the peripheral zone close to the wall of the open field box. The 35 days recording showed changes in the open field behavior of the rats. Both TBI and vehicle treated animals avoided the CZ of the arena, did not spend time in the CZ and when they explored the CZ for the first time, had long latency (Fig. 5A–C). On the other hand, as seen in the heat maps, PG, hMSC or combination treated rats explored the CZ more frequently (Fig. 5A), had lower latency to the first entry to the CZ (Fig. 5B), spent more time in the CZ (Fig. 5C) and entered the CZ more frequently (Fig. 5D).

In the open field arena rats were busy in travelling, rearing and occasional grooming. We did not observe significant difference in their locomotor activities in the open field. The distance traveled or the movement velocity were not significantly different between groups indicating that TBI did not affect the locomotor behavior of the rats (Fig. S4).

#### Immobility in the open field arena

Analysis of other anxiety-like behaviors in the open field showed that the TBI and vehicle treated animals had a shorter latency to become immobile (Fig. 5E), exhibiting anxiety-like behavior in the open field. This behavior improved significantly after the combination treatment as the combination group showed the longest latency to become immobile indicating that they were least anxious in the open field. Combination treatment significantly improved the anxiety-like behavior over the hMSC or PG treated groups (Fig. 5E).

#### Grooming and rearing behavior

TBI and vehicle treated rats showed a significantly increased number of grooming events compared to sham. PG or hMSC treatment did not alter this behavior. However, combination treatment reduced the number of grooming events significantly compared to TBI. The number of grooming events was significantly lower in the hMSC group indicating an improvement in behavior (Fig. 5F).

### Combination treatment improved the TBI induced hyperalgesia

The cold plantar assay showed a significant decrease in the withdrawal latency of the contralateral paw in the TBI or vehicle treated rats compared with sham (Fig. 5G). The combination treatment group had a significant increase in withdrawal latency compared to the control groups (TBI and vehicle). Also, we observed that in the combination treatment group, withdrawal latency increased significantly from the hMSC treated group (Fig. 5B). However, we did not see any significant difference in withdrawal latency in the ipsilateral paw. This observation indicates that the combination treatment helped in recovering from TBI-induced hyperalgesia.

## Discussion

In this study, we have observed significant tissue loss, degeneration of neurons and neuronal fibers in several brain areas 35 dpi, indicating secondary spread of the damage. Presence of Iba1 positive activated microglia, intense astrogliosis and presence of glial scars along with an elevated expression of proinflammatory chemokine CCL20 and IL1-β indicate persistent active inflammation in the brain after TBI in TBI and vehicle treated animals 35 dpi. This is further supported by decreased BDNF in serum after TBI.

For recovery from TBI, it is important to not only restore the damaged tissues but also to prevent the spread of secondary neurodegeneration. Attempts have been made to treat this condition with drugs, but drugs like erythropoietin^4,5^ and progesterone^5,6^ have failed at clinical trials despite their neuroprotective properties. Recently MSCs have become the focus of a regenerative approach in the treatment of TBI. Several investigators have shown the potential of MSCs in cerebral regeneration after TBI. These pluripotent cells are present in a wide variety of body tissues^18,19^ and are easy to isolate and culture and potentially, they can differentiate into many kinds of cells^20–22^. Their immune tolerance^7^, ability to migrate to the site of inflammation^23–25^, and secretion of growth-promoting factors^7–9^ makes them an attractive candidate for regenerative therapies. In our study, we have observed that hMSC treatment improved the behavioral outcome in rats, reduced the brain lesion volume, neuronal death, microglial activation, and CCL20 and IL1- β expression. On the other hand, it increased the expression of BDNF and neurogenesis. Together these observations indicate an overall trend towards the reduction of post-TBI inflammation and increased regeneration. Increased neurogenesis in the hMSC treated animals shows the regenerative potential of these cells but neurogenesis did not increase to the level as observed in the sham animals. Therefore, the expected outcome was not achieved by hMSC treatment alone. On the other hand, the hMSC induced modulations in behavioral, immunological and regenerative outcomes were significantly enhanced by PG treatment prior to hMSC transplantation, i.e., by the combination treatment.

The efficacy of hMSC treatment depends upon a few factors such as the number of administered cells, route of administration, time of administration and the microenvironment of the target tissue. Keeping these factors in mind, we administered an optimum number of cells (1 × 10^6^ cells per animal)^26^ through the intranasal route. Studies have shown that i.v., intra-arterial (i.a.) or intra-cerebral routes have potential disadvantages. Xiong *et al*. showed that i.v. administration of MSCs caused systemic distribution while i.a. administration caused cerebral ischemia and intracerebral administration limits the number of injected cells to a sub-optimal dose^27^. On the other hand, the i.n. route used in this study is the easiest non-invasive delivery route. Bypassing the BBB, the cells enter the cerebral parenchyma through the nasal mucosa and the cribriform plate^28,29^ and reach the brain within 1 hour of administration^30^. In our pilot experiments, we administered DiR-labelled hMSCs via i.v. or i.n. route. We observed significantly higher DiR fluorescence in the brain after i.n. administration compared to the i.v. group (Fig. 1A,B), indicating higher efficacy of this method. In addition, we also observed the presence of the administered cells in the treated brains 35 dpi. The percentage of hMSCs surviving at 35 dpi was not significantly less than the percentage of cells observed at 7 dpi indicating successful transplantation, homing, and survival of cells after i.n. administration. We, therefore, followed this method of hMSC administration throughout this study.

The success of hMSC transplantation therapy also depends on the microenvironment of the recipient tissue. Garcia-Olmo *et al*. have shown that the low success rate of the stem cell transplantation therapy owed to the proinflammatory cytokines and reactive oxygen species secreted by the immune cells in the inflammatory microenvironment of the injured tissue^10,31^. The caspases secreted in this environment caused apoptosis of the transplanted cells, leading to a low success rate of the therapy^32^. Impaired survival and homing efficacy of stem cells due to post-trauma inflammation have been reported by Malcanyi *et al*.^33^. In an attempt to improve the regenerative efficacy of the stem cells in a mouse skin wound model, Geesala *et al*. (2017) transplanted stem cells with or without the oral cyclooxygenase (COX)-2 inhibitor, celecoxib. In this study, they observed increased engraftment and differentiation of stem cells leading to enhanced wound tissue repair when the cells were transplanted with celecoxib^34^. PG, belongs to the group of thiazolidinediones (TZDs) which are synthetic high-affinity ligands of PPARγ; it is a potent anti-inflammatory drug known to reduce the production of inflammatory cytokines after brain injury^35–37^, including IL1-β and IL6^15–17^, tumor necrosis factor α (TNFα), inducible nitric oxide synthase (iNOS), matrix metalloproteinase (MMP)-9 and COX-2 in macrophage, glial cells, and neurons^38^. It has been shown to inhibit CCL20 production, thereby reducing macrophage infiltration and resulting inflammation^14^. It is hypoglycemic^14^, immunomodulatory^16^ and in low doses, it is cardio-protective^13^. It acts on numerous target genes and pathways including the canonical WNT/β-catenin pathway and represses nuclear factor κB (NF- κB) and TNFα, thereby interfering with the pro-inflammatory pathway^39^. In our preliminary study, we observed that PPARγ and CCL20 expression were inversely correlated in the brain. High expression of PPARγ was observed in the brain in the absence of CCL20 expression in sham animals. On the other hand, in TBI animals, PPARγ expression declined with the increased expression of CCL20 (Fig. S3). We also observed PG treatment reduced CCL20 expression post-TBI (Fig. 3). These preliminary observations along with information from published literature prompted us to use PG as the anti-inflammatory drug of choice for this study. We treated the animals with PG following TBI and continued the treatment until hMSC transplantation. This therapeutic strategy enhanced the efficacy of hMSC therapy in decreasing post-TBI inflammation, improving neurotrophic factor secretion and neurogenesis. All these effects were reflected in improved behavioral and sensorimotor responses. The overall treatment outcome of the combination treatment was significantly improved over PG only or hMSC only treatments. Although both hMSC and the combination treatment reduced the lesion and neurodegeneration to a similar extent, the combination treatment was more efficient in reducing the microglial activation in all brain regions examined (Figs 2, S1), even on the contralateral side (Fig. S2). Inflammatory mediators like CCL20 and IL1-β were also significantly reduced by the combination treatment, indicating the efficacy of this strategy over the other treatment options.

Depression and anxiety disorders and locomotor deficits are common and devastating aftereffects of TBI. In this study, we did not observe motor deficits in the rats 35 dpi as there was no significant change in their locomotor activity in the open field (Fig. S4). Although generally considered psychological, anxiety and depression may have neurobiological underpinnings. The open field test in rodents is a simple, yet promising, test to evaluate such neurobehavioral aspects of TBI. In an open field arena, rats typically prefer to be close to the walls, a behavior called thigmotaxis. They tend to avoid the open, well-lit area of the center which is a novel, stressful environment to the animal while making occasional exploratory trips to the center area. When the animals are less anxious they tend to spend more time in the open center zone and also decrease the latency to enter this area (anxiolytic activity). Jones *et al*. (2008) showed that 1 to 3 months after TBI induced by fluid percussion injury (FPI), rats showed anxiety-like behavior by exhibiting reduced entry and reduced time spent in the center area of an open field arena^40^. Recently, Kim *et al*. have shown increased activity in the center zone of rats treated with the synthetic estrogen, 17α-ethynylestradiol-3-sulfate, after FPI-induced TBI^41^. In line with these reports, our findings indicate that TBI and vehicle treated rats showed significant anxiety-like behavior and this behavior improved in the combination treatment group over other treatment groups. The rats with combination treatment showed lower latency to enter the CZ, they spent more time in the CZ and entered the zone more frequently indicating anxiolytic behavior. They also exhibited the longest latency to first immobility and the least grooming. All these behaviors indicated that the functional recovery was best in the combination treated rats.

Sensory hypersensitivity and persistent pain, including headache, nociceptive pain, and neuropathic pain are somatic symptoms of TBI^42^. Pain has been reported as a secondary complication of TBI^43,44^. Very few models are available that describe post-injury pain and its mechanism^45,46^. In the current study, we have shown increased thermal pain sensitivity (decreased withdrawal latency to noxious cold exposure) in the contralateral paw in rats after TBI. This indicates the involvement of the central sensorimotor pathway in this phenomenon. Following TBI, synthesis and release of inflammatory cytokines and pro-nociceptive mediators from activated microglia have been reported^47^. Rowe *et al*. implicated glial activation, central and peripheral inflammatory mediators and T_reg_ dysregulation as potential causes of TBI-induced mechanical hyperalgesia^42^. In our study, combination treatment has shown to reduce the hyperalgesia to the normal level. It is possible that this mechanism is correlated to the reduction of cerebral neuroinflammation, tissue regeneration and re-establishment of the neural connections by the combination treatment after TBI. Further investigation is needed to elucidate the actual mechanism of combination treatment induced alleviation of sensorimotor response to noxious cold sensation after TBI. Regardless of the mechanism, our observations strongly suggest a behavioral and sensorimotor recovery in the combination treatment group after TBI.

We observed that hMSC or combination treatment increased GFAP immunoreactivity, although no glial scar formation was observed in the treated groups. The astrocytes in these groups maintained their territory all over the brain. At the same time, CCL20 decreased in these groups indicating the reduced inflammatory status of the brain. Astrocytes are an important cellular component in maintaining the structure and functions of CNS. They are essential for neuronal survival^48^. Reactive astrogliosis is indicative of injury or insult to the brain^49^. They can be neuroprotective or neurotoxic^49,50^. Following TBI, astrocytes help in restricting the spread of damage by forming glial scars^20^. It has been shown that MSCs release chemokines which activate surrounding astrocytes facilitating tissue repair^20,51^. Following administration after TBI, MSCs have migrated to a distant location from the lesion site and differentiated into neurons and astrocytes improving motor functions^20^. It is possible that in our study, astrocytosis in the hMSC or combination treated animals is actually neuroprotective or regenerative. More research is needed to elucidate the exact role played by the astrocytes under the present circumstances.

Neurotrophic factors are associated with reactive processes occurring as a result of lesions in the CNS. BDNF is a neurotrophic factor which has been shown to be associated with post-TBI depression and cognitive dysfunction^52,53^. It is also involved in neuronal survival and synaptic plasticity^54,55^. Following TBI, a decrease in serum BDNF is correlated with injury severity^56^ and poor recovery^53^. Recently, it has been shown that exogenous BDNF helps in mitigating neuronal metabolic defects and thereby improves neuronal survival after mechanical injury^57^. Protective effects of exogenous BDNF against neuronal apoptosis following mild ischemic brain injury has been shown by Galvin and Oorschot (2003)^58^, whereas the commercially available drug, simvastatin, increased hippocampal BDNF level and neurogenesis in a rat model of TBI^59^. It is possible that neuropathological and behavioral changes after TBI observed in this study were mediated, at least partially, through BDNF. One of the major mechanisms by which hMSCs exert their effects in CNS repair and regeneration is by secreting neurotrophins^52,60,61^. Following transplantation, these cells may directly influence neuronal repair or stimulate the glial cells to secrete neurotrophins like BDNF or Nerve Growth Factor. On the other hand, the interaction between glial cells and hMSCs may lead to neurotrophin release and subsequent neuronal repair^61,62^. In this study, we observed that combination treatment, which showed the best recovery of histological, behavioral and sensorimotor parameters, also had significantly increased BDNF levels in serum as compared to the TBI or vehicle groups. Thus, it clearly indicates at least partial involvement of BDNF in the combination treatment induced recovery mechanism.

In stem cell therapy of brain injury, a key step in recovery is neuro-regeneration. *In vitro* differentiation of embryonic stem cells to neural cells has been shown by Guan *et al*.^63^. Mahmood *et al*. have shown that hMSC injected in the rat after TBI promotes tissue repair through neurogenesis and synaptogenesis^64^. Adults have two major neural stem cell niches, the SVZ and the dentate gyrus^65^. We observed that both hMSC and combination treatment enhanced neurogenesis in these regions. But the extent of neurogenesis reached the level of the sham animals only in the combination therapy group, which clearly showed the efficacy of this therapeutic strategy. Since BDNF has a potent role in neuroprotection and neurogenesis^66^, it is possible that hMSC induced neuroprotective and neurogenic effects were mediated, at least partially, through BDNF. BDNF along with other neurotrophic factors has been shown to utilize the canonical β-catenin pathway to promote hMSC neurogenesis and synaptogenesis^67^. The specific mechanism of PPARγ activation in the improvement of hMSC functioning and hMSC-induced neurogenesis needs further investigation.

**Figure 6:**
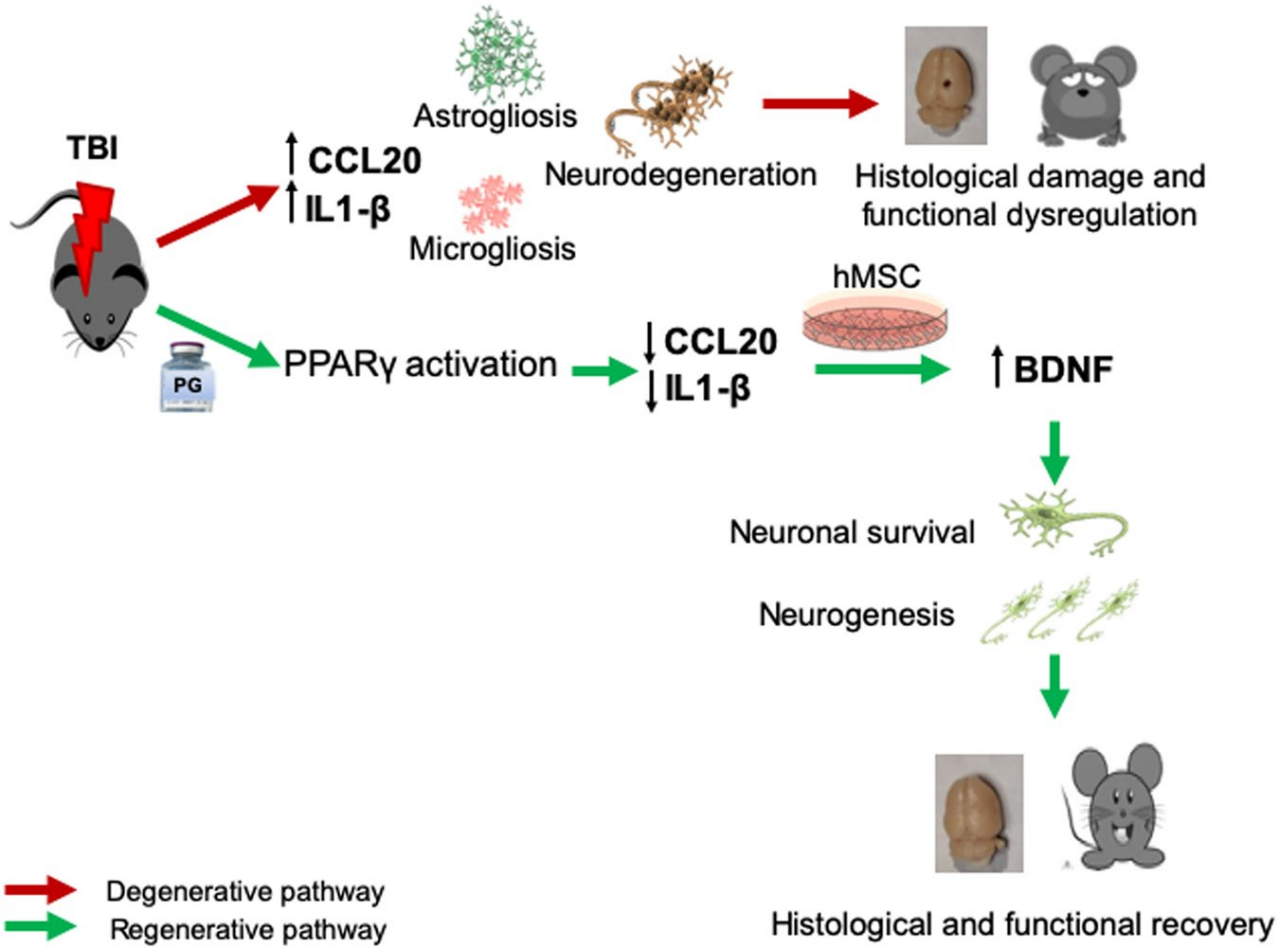
Schematic representation summarizing the effect of Pioglitazone (PG) and hMSC combination treatment on improving outcomes in rats after traumatic brain injury (TBI). TBI induces neurodegeneration and evokes inflammatory reactions including elevated cytokines CCL20 and IL1-β, microgliosis and astrogliosis. These lead to histological and functional deficits. PG treatment after TBI activates PPARγ and reduces CCL20 and IL1-β. In the reduced inflammatory microenvironment hMSCs increased BDNF secretion which at least partially improves the histological and functional recovery. CCL20, Chemokine ligand protein 20, IL1-β, Interleukin 1 beta, hMSC, human mesenchymal stem cells, BDNF, brain-derived neurotrophic factor.

In summary, as shown in Fig. 6, FPI induced injury in rat brains with significant tissue loss and neurodegeneration which persisted at 35 dpi. TBI in rats induced microglial and astroglial activation, increased secretion of proinflammatory cytokines and caused behavioral and sensorimotor deficits. PG reduced neuroinflammation in the brain by decreasing inflammatory cytokine production prior to hMSC transplantation. Reduction of local cerebral inflammation improved the efficacy of transplanted hMSC which was evident by increased neurogenesis, reduced anxiety-like behavior and reduced pain sensation in combination treated rats. Possibly, in a reduced inflammatory microenvironment, hMSCs helped in histological and behavioral recovery through enhanced production of neurotrophic factors like BDNF. Thus, in this study, we have shown that using PG before hMSC transplantation is a better therapeutic strategy for treating TBI compared to hMSC transplantation alone.

## Methods

### Animals and TBI induction

All animal procedures were conducted in accordance with the NIH Guide for the Care and Use of Laboratory Animals and approved by the Institutional Animal Care and Use Committee of the University of South Florida. Forty male Sprague-Dawley rats (Envigo, USA) weighing 250 to 300 g were housed in a climate-controlled facility with food and water available *ad libitum*. Animals in this study were divided into 6 groups: 1. Sham (n = 8), 2. TBI (n = 6), 3. Vehicle treated (TBI + DMSO, n = 5), 4. PG treated (TBI + PG, n = 8), 5. hMSC treated (TBI + hMSC, n = 6), 6. Combination treatment (Combo.) (TBI + PG + hMSC, n = 7).

Animals were anesthetized using a mixture of ketamine (90 mg/kg) and xylazine (10 mg/kg) (intraperitoneal, i.p.). Carprofen (5 mg/kg, subcutaneous, s.c., pre-operative and post-operative up to 48 h) and buprenorphine (5 mg/kg, s.c., pre-operative) were administered to all animals. Body temperature was monitored and maintained using a heating pad during the entire surgical procedure. Using a stereotaxic frame, a 1 mm diameter craniotomy was performed with a 0.7 mm drill bit, 2 mm lateral and 2.3 mm caudal to the bregma on the right side of the midline^11^. In case of accidental disruption of the meninges, the animal was excluded from the study. A female luer-lock hub was implanted at the craniotomy site, secured with dental cement and connected to fluid percussion injury (FPI) device. An impact ranging from 2.5–3.0 atm. was administered. Sham animals underwent all surgical procedures except the impact delivery. The luer-lock was detached, the craniotomy hole sealed with bone wax, and the scalp was sutured. Rats were returned to their home cages and allowed to recover for 35 days prior to subsequent experiments.

### Drug and cell administration

Pioglitazone hydrochloride (PG) (Tocris, Inc.) dissolved in dimethyl sulfoxide (DMSO) at a dose of 2 mg/kg in 100uL or equal volume of vehicle was injected i.p. once a day for 5 days after TBI.

hMSCs were obtained from the Institute for Regenerative Medicine, Texas A&M Health Science Center. Cells were cultured in α Minimum Essential Medium (αMEM) (Gibco, cat#12561-056) supplemented with 16.5% FBS, 2mM L-glutamine and 1% penicillin and streptomycin at 37 °C and 5% CO_2_. At 90–95% confluence, cells were detached, washed, and resuspended in sterile phosphate buffered saline (PBS). The final volume of the cell suspension was adjusted so that 1 × 10^6^ live cells were present in 100 µL of cell suspension. On day 5 post impact, hyaluronidase was applied to each nostril (100U) and after 30 min hMSCs were instilled through the nostrils (50 µL/nostril) under isoflurane anesthesia. Rats were allowed to recover on a heating pad and returned to their home cage. hMSCs labeled with 1 µg/mL DiR (XenoLight DiR, Caliper Lifesciences) were washed and resuspended in the required volume of sterile PBS. Rats were injected with 1 × 10^6^ hMSCs either through the tail vein (i.v.) in 200 µl or by the i.n. route 50 µl per nostril. Animals were imaged after 7 days with the IVIS system using 710 nm excitation and 760 nm emission. Radiant efficiency (photons per second per square centimeter per steridian divided by microwatts per square centimeter ((p/s/cm^2^/sr)/(µW/cm^2^)) of the region of interest (ROI) was plotted for each organ and each method of administration.

### Open field test (OFT)

After 7 days of acclimation and gentle handling, baseline activity in the open field arena (90 cm (W) × 90 cm (D) × 40 cm (H) cm enclosure) was recorded for 10 min using Noldus Ethovision XT 10 software. The OFT was repeated for 10 min on 35 dpi. Rats were gently placed in the center of the arena and allowed 2 min to acclimatize in the arena before the start of the recording. The arena was cleaned with 70% ethyle alcohol and dried between rats. Activities in the peripheral and central zone were analyzed. Grooming and rearing behaviors were analyzed independently by 3 people blinded to the experimental conditions and averaged.

### Cold plantar assay

The Cold plantar assay was performed to assess sensorimotor behavior. On 35 dpi rats were held gently in the lap by one experimenter with the hind paws hanging freely. A small portion of powdered dry ice compacted in the shape of a stick was pushed out from the cylinder of a syringe with the plunger and wrapped in a nitrile glove. The wrapped dry ice was touched to the plantar of the freely hanging hind paws, one paw at a time and the time of paw withdrawal was recorded. Care was taken that the paw movement was not hindered by any means. An additional group of naïve animals served as controls.

### Euthanasia, tissue harvest, and processing

On 35 dpi animals were deeply anesthetized with ketamine (75 mg/kg) and xylazine (7.5 mg/kg). Blood was collected and rats were perfused with 0.9% saline followed by 4% paraformaldehyde (PFA) in 0.1 M phosphate buffer (pH 7.4). The brains were post-fixed in 2% PFA, saturated with sucrose solution, frozen, and 30 μm coronal cryo-sections were generated.

### Thionin and Fluorojade staining

Sections were treated with 1.25% thionin acetate solution (Sigma Aldrich, USA) for 45 seconds followed by rinsing with deionized water for 1 min., dehydrated through graded ethanol, cleared with xylene, and coverslipped with DPX mounting medium (Electron Microscopy Sciences, Ft. Washington, PA). For fluorojade (FJ) staining^68,69^ slide-mounted sections were hydrated in graded ethanol and then oxidized with 0.06% KMnO4 solution for 15 minutes and stained in a 0.001% solution of FJ (Histochem, Jefferson, AR) in 0.1% acetic acid for 30 min. Slides were rinsed, dried at 45 °C for 20 min, cleared with xylene, and coverslipped using DPX mounting medium.

### Immunohistochemistry

For immune-peroxidase staining, sections were incubated in 3% hydrogen peroxide for 20 min, washed with PBS and heated in antigen unmasking solution (1:100; Vector Laboratories Inc., Burlingame, CA) for 40 min at 90 °C, cooled, permeabilized for 1 h in permeabilization buffer (10% host serum, 0.1% Triton X-100 in PBS) and incubated overnight at 4 °C with primary antibody in antibody solution (5% host serum, 0.05% Triton X-100 in PBS). Next day, sections were washed with PBS, incubated sequentially with biotinylated secondary antibody (2 h, room temperature (RT)) and avidin-biotin complex mixture (ABC,1:100; Vector Laboratories, Inc., Burlingame, Ca) (1 h, RT) and developed using DAB/peroxide solution (Vector Laboratories, Inc.). After 3 washes, sections were dried, dehydrated, cleared with xylene and coverslipped with DPX. For fluorescence immunohistochemistry, no peroxide blocking was performed. After primary antibody incubation, sections were incubated with fluorescent secondary antibody, washed with PBS, dried and cover slipped with vectashield anti-fade mounting medium with DAPI. Detailed information on antibodies used in this study is shown in Table S1. Sections were viewed with an Olympus IX71 microscope using appropriate filters. Images were captured using an Olympus DP70 imaging system. The low magnification (4x) collages of the entire brain sections were taken and processed with a Keyence BZ-X800 microscope and associated software (Keyence America).

### Enzyme Linked Immunosorbent Assay (ELISA)

Serum samples were thawed on ice for ELISA development using Picokine rat BDNF ELISA kit from myBiosource (San Diego, USA, Cat# MBS175935) following manufacturer’s instruction. Briefly, standards or samples were added to wells of a 96 well ELISA plate pre-coated with anti-BDNF antibody and incubated overnight at 4 °C. The plate was washed, and incubated sequentially with biotinylated anti-rat BDNF antibody for 1 h, avidin-biotin-peroxidase complex for 30 min and color development reagent for 25 min at room temperature. Reactions were stopped with 2N H_2_SO_4._ The absorbance readings were taken at 450 nm using a Synergy H4 hybrid reader (BioTek). Serum BDNF concentration was expressed as pg/mL.

### Image analysis and quantitation

Cell count or intensity was calculated using the NIH Image J software. Images (100x or 200x) were taken at the same exposure and digital gain settings for a given magnification for all the sections in order to eliminate differential background intensity and/or false positive signal. The RGB channels of fluorescent images were split and either the red or green channel was used for Quantitation. The bright field images were converted to grey scale. The brightness and contrast were adjusted to discard the noise pixels. The threshold of the binary images was adjusted between 0 and 255 to highlight all positive cells to be counted. In the set measurement tool, the particle sizes were adjusted to exclude the small noise pixels or the large clumps from the count. Circularity was adjusted between 0 and 1 to discard any cell fragments, cell processes, or tissue aggregates that can create false results. The same specifications were used to quantitate across the board. The number of cells was counted using Analyze > Particle tool. The fluorescence intensity (integrated density) was measured using Analyze > Measure tool. For lesion volume measurement, the scale was calibrated using the set scale tool, the horizontal and vertical diameters of the lesion area were measured and averaged. The lesion area from each section was calculated using the average diameter and multiplied by the section thickness (30 µ) to calculate the lesion volume for each section. The values were then added up to obtain total lesion volume from each brain and as expressed as mm^3^.

### Statistical analysis

All data are presented as mean ± Standard Error of Mean (S.E.M.). Statistical significance was evaluated by one-way ANOVA with a Tukey’s or Fisher’s post-hoc test or student’s t-test. A p value of less than 0.05 was considered statistically significant for all comparisons.

## Supplementary information

Supplementary Info
