# Supplementary material for: Pioglitazone treatment prior to transplantation improves the efficacy of human mesenchymal stem cells after traumatic brain injury in rats
Source: Sci Rep. 2019 Sep 20;9:13646. doi: 10.1038/s41598-019-49428-y (PMC6754424; doi:10.1038/s41598-019-49428-y)
Supplement: Supplementary file 1 — Supplementary Info [file 41598_2019_49428_MOESM1_ESM.pdf]

Supplementary information.

Title: Pioglitazone treatment prior to transplantation improves the efficacy of human mesenchymal stem cells after traumatic brain injury in rats.

Mahasweta Das[1,2], Karthick Mayilsamy [1,2], Xiaolan Tang[1,2], Jung Yeon Han[1,2], Elspeth Foran[1,2], Alison E. Willing[4], Shyam S. Mohapatra[1,3] and Subhra Mohapatra[1,2*]

[1]James A. Haley Veterans Hospital, [2]Department of Molecular Medicine, [3]Department of Internal Medicine, [4]Department of Neurosurgery and Brain Repair, University of South Florida College of Medicine, Tampa, FL 33612, USA.

Contact: * Corresponding author

Email: smohapa2@health.usf.edu

Table S1. Primary antibodies and corresponding secondary antibodies used for immunohistochemistry in the experiment.

Primary antibody	Source	Catalogue number	dilution	Secondary antibody	Source	Catalogue number	dilution	development
Anti-human nuclear antigen antibody (Clone 235-1)	Abcam, Cambridge, MA	Ab191181	1:100	Alexafluor 594-anti rabbit	Invitrogen	A11008	1:1000	Fluorescence
rabbit anti-CCL20	Abcam, Cambridge, MA	ab25123	1:1000	Biotinylated goat anti-rabbit Vector Laboratories Inc., Burlingame, Ca	Vector Laboratories Inc., Burlingame, Ca	BA-1000	1:400	DAB
rabbit anti-Iba1	Waco Chemicals, CA	019-19741	1:1000	Alexafluor 594-anti rabbit	Invitrogen	A11008	1:1000	Fluorescence
Chicken anti-GFAP	Invitrogen	AB5541	1:1000	Alexafluor 488- anti chicken	Invitrogen	A11039	1:1000	Fluorescence
rabbit anti-doublecortin (DCX)	Abcam	Sc-8066	1:250	Alexafluor 488-anti rabbit	Vector Laboratories Inc., Burlingame, Ca	A11008	1:1000	Fluorescence
Rabbit anti PPARγ	Abcam	Ab ab19481	1:1000	Biotinylated goat anti-rabbit Vector Laboratories Inc., Burlingame, Ca	Vector Laboratories Inc., Burlingame, Ca	BA-1000	1:400	DAB

Supplementary figures:

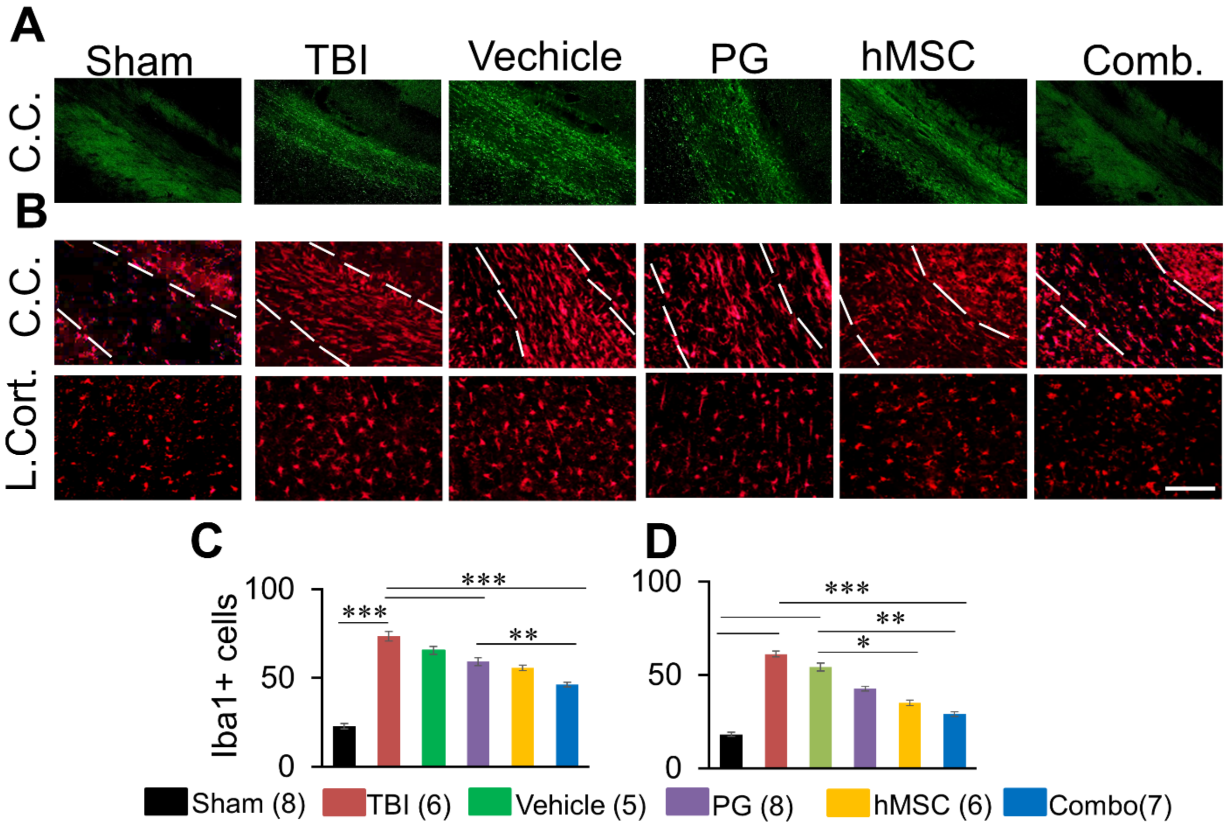

Figure S1. Effect of PG + hMSC combination treatment on TBI induced neurodegeneration and microgliosis. A, Fluorojade expression in the corpus callosum. B. Representative immunofluorescence images showing the Iba1 positive microglia in different brain regions of the ipsilateral side. White dotted lines indicate the boundaries of the corpus callosum. Scale bar 100μ. B, Average number of Iba1 positive microglia (mean ± SEM) in ipsilateral corpus callosum (B) and lateral cortex (D). C.C., Corpus callosum, L.Cort., lateral cortex, PG, pioglitazone, hMSC, human mesenchymal stem cells, Comb., PG + hMSC combination treatment. Numbers in the parentheses indicate number of animals in each group. * $p < 0.01$. ** $p<0.001$, *** $p<0.0001$.

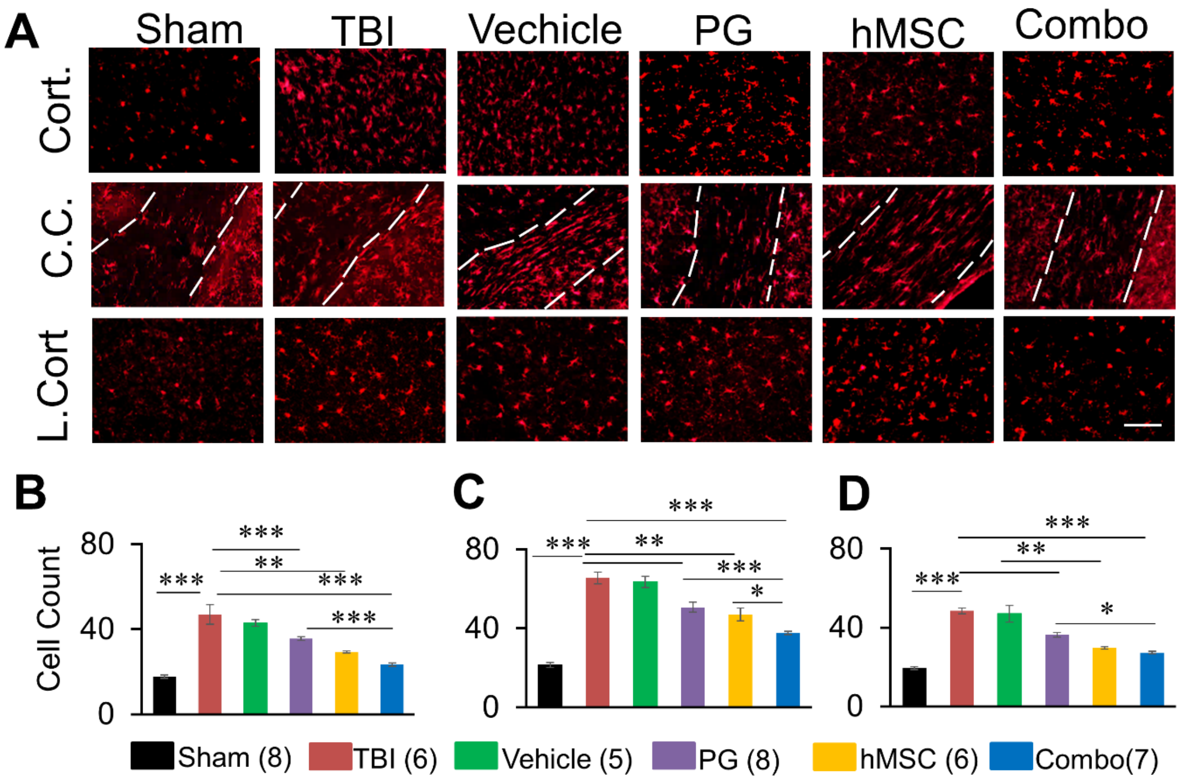

Figure S2. Effect of combination treatment on microglial activation. A, Representative immunofluorescence images showing the Iba1 positive microglia in different brain regions of the contralateral hemisphere. White dotted lines indicate the boundaries of the corpus callosum. Scale bar 100µ. B -D, Average number of Iba1 positive microglia (mean ± SEM) in ipsilateral cortex (B), corpus callosum (C) and lateral cortex (D). C.C., Corpus callosum, L. Cort., lateral cortex, PG, pioglitazone, hMSC, human mesenchymal stem cells, Combo., PG + hMSC combination treatment. Numbers in the parentheses indicate number of animals in each group. * p< 0.01. **p<0.001, ***p<0.0001.

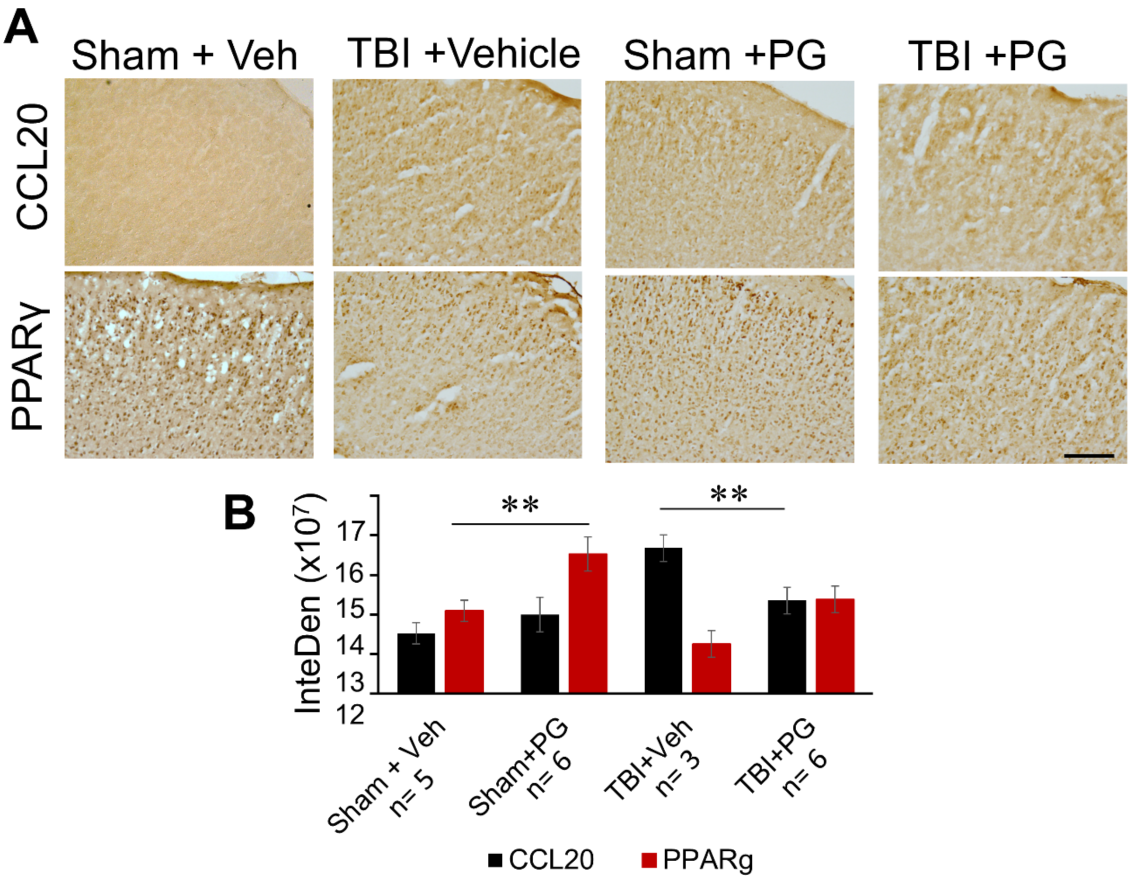

Figure S3. Pioglitazone treatment reduces CCL20 expression in the brain 48h after TBI. A, Photomicrographs showing the expression of CCL20 or PPARγ in the cortex. Scale bar 200μ. B, Immunoreactivity (mean ± SEM) analyses show that pioglitazone treatment partially reduced the CCL20 expression in the cortex. Pioglitazone treatment increased PPARγ expression in the cortex of sham animals. **p<.001.

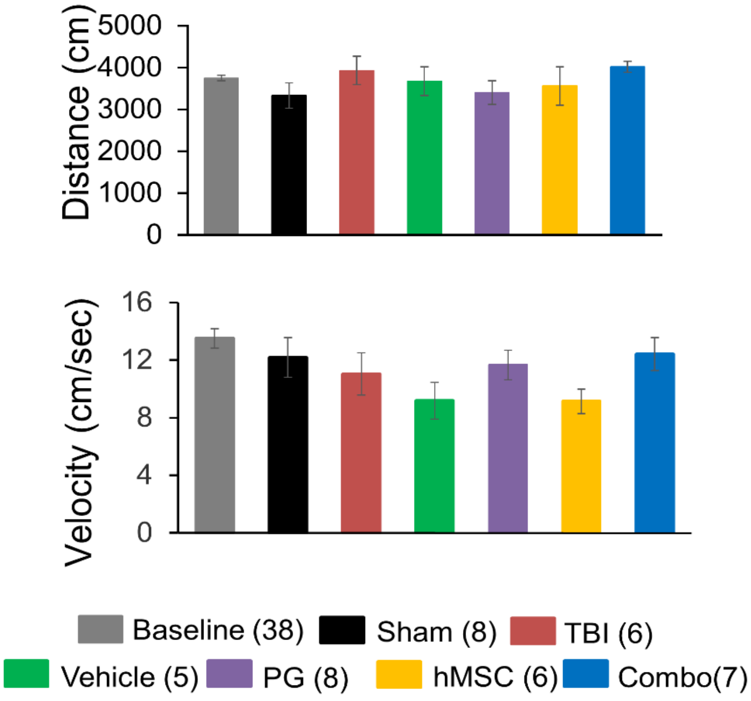

Figure S4. Total distance traveled in the Open field arena by rats under different experimental conditions. Histograms show the average values (mean ± SEM) of total distance traveled (A) by the rats or mean velocity of movement (B) in the open field arena 35 days post TBI. No significant difference in the distance traveled or the movement velocity was observed. Numbers in the parentheses indicate number of animals in each group
